# Case report of vaginal erosion and recurrence of stress urinary incontinence due to shallow placement of tension-free vaginal tape

**DOI:** 10.1186/s12894-022-01016-5

**Published:** 2022-04-22

**Authors:** Xiao Huang

**Affiliations:** Department of Urology, The First Affiliated Hospital, School of Medicine, Zhejiang University, No. 79 Qingchun Road, Hangzhou City, 310003 Zhejiang Province People’s Republic of China

**Keywords:** Stress urinary incontinence, Recurrence, Tension-free vaginal tape, Tape erosion, Complications

## Abstract

**Background:**

Suburethral sling with tension-free vaginal tape (TVT) has become a popular treatment for stress urinary incontinence (SUI). Erosion of the tape into the vaginal is rare. Very few patients present with vaginal tape erosion and recurrence of SUI.

**Case presentation:**

A 49-year-old female patient with stress urinary incontinence was treated with a retropubic suburethral TVT sling. 2 months later, recurrent symptoms of SUI developed. 6 months later, the patient complained of repeated vaginal discharge and foreign body sensation. Body physical examination revealed a 1-cm-long tape extrusion at the left anterior vaginal wall beside the midline. Cystourethroscopy revealed no urethral mesh erosion. Surgical removal of the extrusion tape revealed that the left arm of the tape was in the vaginal mucosa layer rather than between the whole thickness of the vaginal mucosa and urethral. The tape around the urethral was dissected and removed. A new retropubic tape was placed simultaneously. At the 8-months follow-up after surgery, the patient was continent without tape vaginal exposure.

**Conclusions:**

Vaginal mesh erosion should be considered in a patient who presents with sustained vaginal discharge after being treated with a suburethral sling. It is important to place the tape between the whole thickness of the vaginal mucosa and the urethral in SUI surgery. A shallow placement of the tape may lead to vaginal tape erosion and recurrence of SUI. These complications can be avoided by following the correct manipulation procedure and referring to the tissue layer anatomy.

## Background

Stress urinary incontinence (SUI) is a major urological problem as many as 25% of women older than 20 years have SUI [1].The retropubic tension-free vaginal tape (TVT) procedure has become the standard of care for SUI with demonstrated long-term efficacy and low complications since its introduction in 1996 by Ulmsten et al. [2]. Vaginal erosion after TVT or transobturator tape placement is a rare complication with an incidence ranging from 0.2 to 22% [3, 7].Tape vaginal erosion with SUI recurrence together is extremely rare [4, 5]. We recently experienced a case of rapid SUI recurrence and later vaginal tape exposure because of the shallow placement of the tension-free vaginal tape.

## Case presentation

A 49-year-old woman was referred to our center because of vaginal tape exposure for 2 weeks and recurrent SUI symptoms for 4 months. The patient had a history of a retropubic TVT sling procedure performed at 6 months ago due to SUI at a local hospital. 2 month later after the TVT surgery, the patient had recurrent SUI symptoms, such as urine leakage when sneezing and coughing. After physical examination and symptoms estimation, no special treatment was recommended to the patient by the physician of the local hospital. 6 months after surgery, the patient had aggravating urine leakage symptoms and repeated vaginal discharge and foreign body sensation. Vaginal tape exposure was found by her male partner for dyspareunia 2 weeks before the patient came to our center. Her medical history was significant for hypertension history for 2 years. Her blood pressure was well controlled with candesartan. The patient had once natural delivery and was in menopausal status before TVT surgery.

**Fig. 1:**
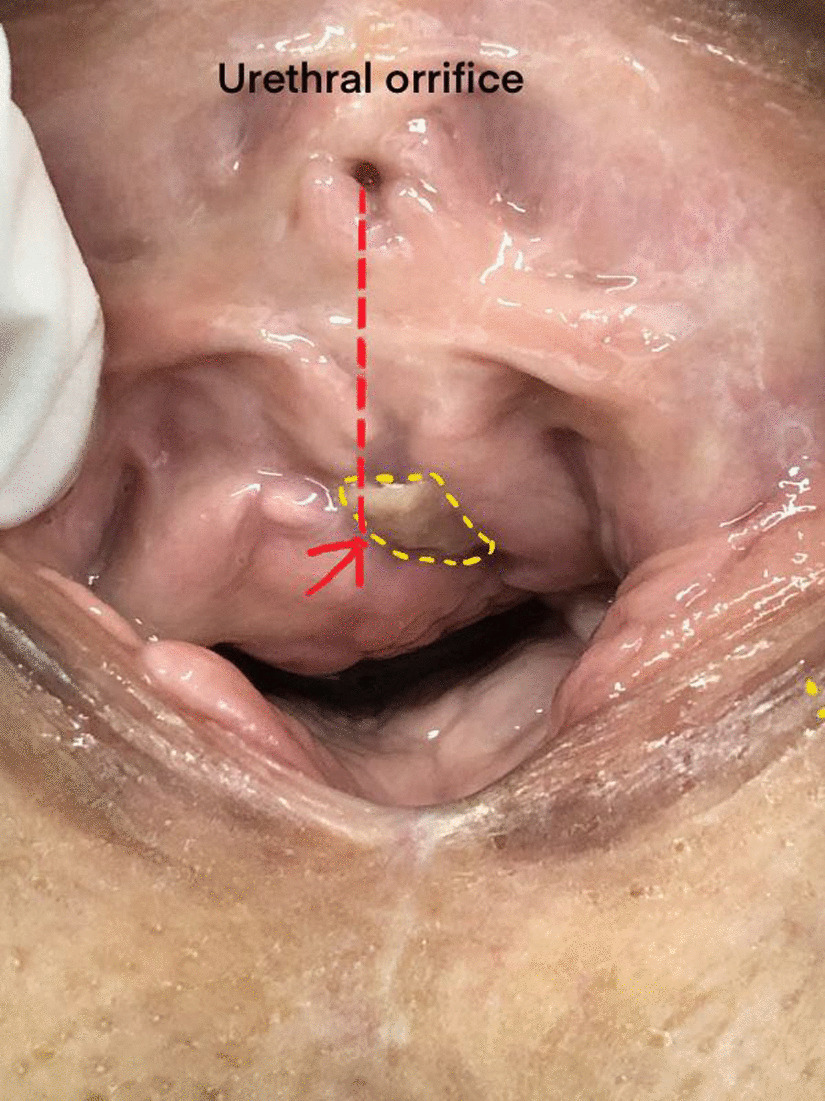
Body physical examination revealed a 1-cm-long tape (yellow circle contour) extruding from the left anterior vaginal wall beside the midline (red arrow)

**Fig. 2:**
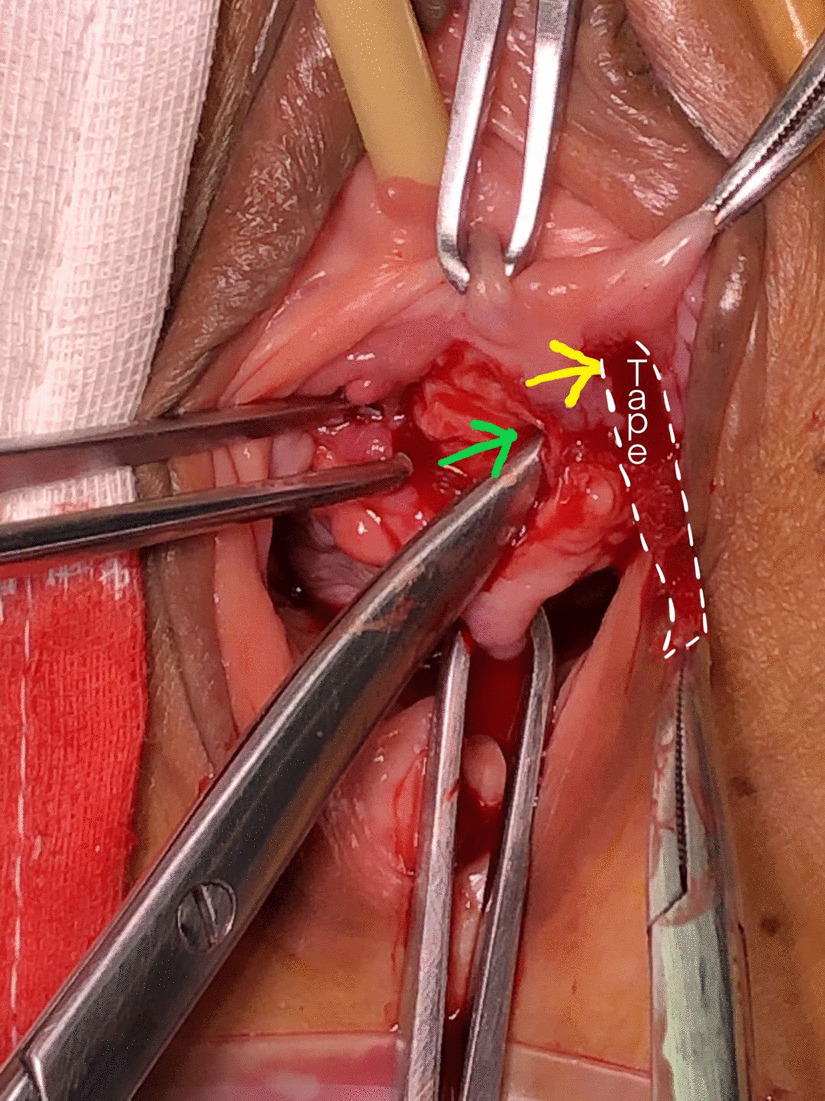
Transvaginal periurethral dissection was performed. Surgical removal of the extruded tape revealed that the left arm of tape (white contour) was wrongly put in the vaginal mucosa layer (yellow arrow) rather than between the whole thickness of vaginal mucosa and urethral layer (green arrow)

**Fig. 3:**
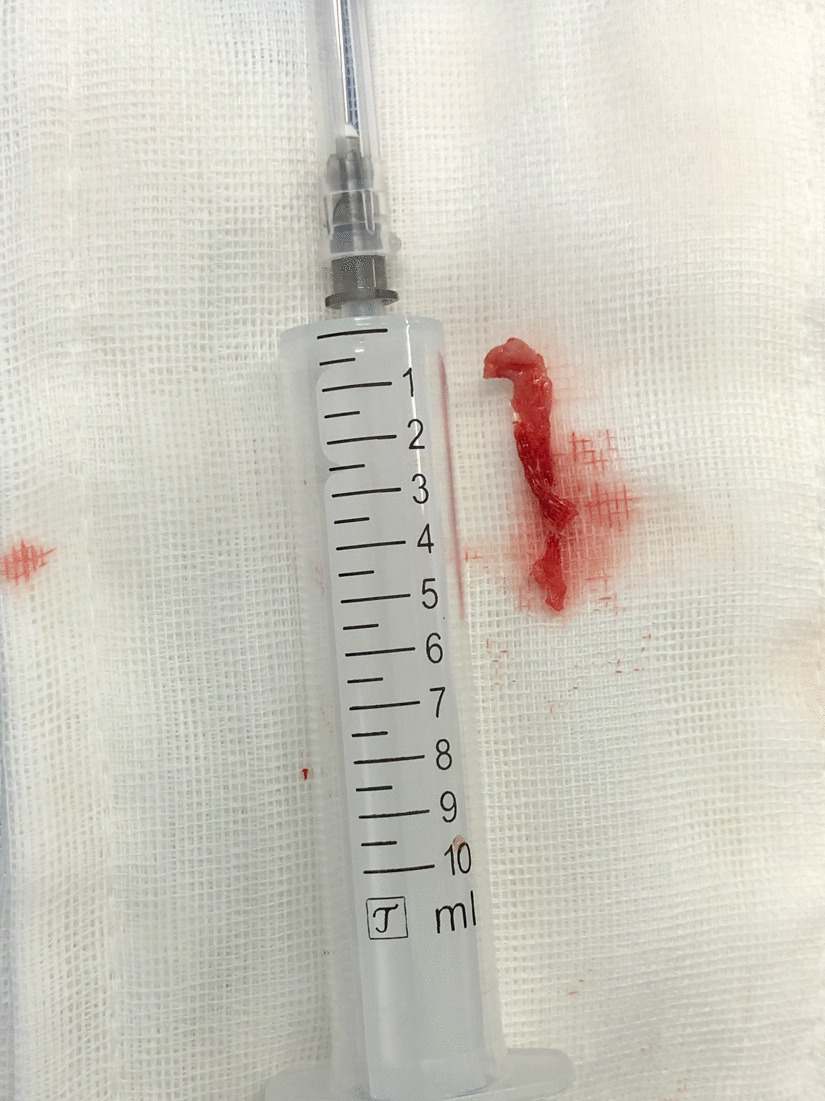
A 5-cm-long tape around the urethral was dissected and removed

Urinalysis and vaginal secretions were negative. Body physical examination revealed a 1-cm-long tape extruding (yellow circle contour) from the left anterior vaginal wall beside the midline (Fig. 1, red arrow). Cystourethroscopy revealed no urethral tape erosion. Urodynamic examination confirmed the SUI diagnosis. A 2-cm-long longitude incision in the anterior vaginal wall was made. Transvaginal periurethral dissection was performed. Surgical removal of the extruded tape revealed that the left arm of the tape was wrongly put in the vaginal mucosa layer(Fig. 2, yellow arrow) rather than between the whole thickness of vaginal mucosa and urethral (Fig. 2, green arrow). A 5-cm-long tape around the urethral was dissected and removed (Fig. 3). A new retropubic tape was placed simultaneously. At the 8-months follow-up after surgery, the patient was continent without tape vaginal exposure.

## Discussion and conclusions

Although the TVT sling procedure either by retropubic or transobturator way is been demonstrated to be a safe and effective surgery for SUI, there have been reports of complications. The most common complications are de novo urge incontinence and voiding dysfunction [3, 4, 6]. Vaginal tape erosion/exposure/extrusion is a rare complication with an incidence ranging from 0.2 to 22% [3, 7].Tape vaginal erosion with SUI recurrence together is extremely rare [4, 5]. The exact reasons for vaginal tape erosions remain uncertain, but possible explanations include wound infection, excessive sling tension, small mesh pore size, poor mesh incorporation, radiotherapy, postoperative vaginal atrophy, and premature sexual intercourse after operation [7–9]. Risk factors for erosion are the length of vaginal incision > 2 cm, recurrent vaginal incision for postoperative complications, and previous pelvic organ prolapse or incontinence surgery [7]. In our case, none of the above reasons can explain the mechanism of rapid SUI recurrence and later tape erosion. We verified in surgery that the right arm was in the proper layer of the vaginal wall and had no erosion. The left arm of the tape, however, was wrongly put in the vaginal mucosa layer rather than between the whole thickness of vaginal mucosa and urethral. Thus, shallow placement of the tape may cause rapid recurrence of SUI and vaginal tape erosion. In order to get the correct anatomy for the tape placement, we usually inject 10–20 ml saline into the vaginal mucosa. The incision should be deep enough to separate the whole thickness of the vaginal wall. Blunt dissection is recommended by using tissue scissor. It`s important to make sure that the bilateral tissue tunnels for the tape placement are in the same layer of the vaginal wall. After dissection of the vaginal mucosa, the urethral should be in integrity.

Symptoms of vaginal tape erosion include sustained vaginal discharge, vaginal bleeding, dyspareunia of the patient or her partner [8–10]. Some patients may have no symptoms, so a careful vaginal examination is needed after surgery for follow-up. Vaginal tape erosion without other problems can be treated in a conservative way, such as prointravaginal estrogen administration [5, 11]. When conservative treatments fail, partial or complete tape removal is recommended [5, 11, 12]. For the correction of recurrent SUI, retropubic TVT tape is recommended for high priority with encouraging success [13]. Therefore, after partial removal of periurethral tape, we put another retropubic vaginal tape. At the 8-months follow-up after surgery, the patient was continent without tape vaginal exposure.

For the technique of TVT either by retropubic or transobturator pathway, it is important to place the tape between the whole thickness of the vaginal mucosa and the urethral in SUI. A shallow tape placement may cause rapid SUI recurrence and the vaginal tape erosion. These complications can be avoided by following the correct manipulation procedure and referring to tissue layer anatomy.

## Data Availability

The datasets used and analysed during the current study are available from the corresponding author on reasonable request.

## Abbreviations

SUI: Stress urinary incontinence
TVT: Tension-free vaginal tape
